# Metabolomic profiling reveals dynamic lipid reprogramming during adipogenesis in 3T3-L1 cells

**DOI:** 10.3389/fmolb.2026.1821798

**Published:** 2026-05-26

**Authors:** Nanping Shi, Zeyi Huang, Jianan Liu, Wuping Sun, Jian Li

**Affiliations:** 1 Shenzhen Maternity and Child Healthcare Hospital, Women and Children’s Medical Center. Southern Medical University, Shenzhen, Guangdong Province, China; 2 Department of Clinical Laboratory Medicine, Fifth Affiliated Hospital, Southern Medical University, Guangzhou, China; 3 Shenzhen Luohu People’s Hospital, The Third Affiliated Hospital (The Affiliated Luohu Hospital) of Shenzhen University, Shenzhen University, Shenzhen, China

**Keywords:** 3T3-L1 cells, adipocyte differentiation, adipogenesis, metabolomic, obesity

## Abstract

**Objective:**

Adipogenesis, the process of adipocyte differentiation, plays a central role in obesity development. However, the molecular mechanisms underlying adipogenesis and its regulation remain incompletely understood. This study aimed to investigate metabolomic changes during adipocyte differentiation in 3T3-L1 cells and elucidate the associated molecular mechanisms, with potential implications for obesity-related metabolic diseases.

**Methods:**

Murine 3T3-L1 pre-adipocytes were cultured and induced to differentiate into mature adipocytes over an 8-day period. Samples were collected at pre-differentiation (Pre), 4-day differentiation (Middle) and 8-day differentiation (Mature) stages. Metabolites were extracted using hydrophilic and hydrophobic methods and analyzed by ultra-performance liquid chromatography coupled with mass spectrometry. Data analysis employed kyoto encyclopedia of genes and genomes (KEGG) pathway enrichment analysis to identify differential metabolites and their associated metabolic pathways.

**Results:**

Significant differences in metabolite profiles were observed among Pre, Middle and Mature. We identified 170 differential metabolites between Pre and Middle (164 increased, 6 decreased), 246 differential metabolites between Pre and Mature (223 increased, 23 decreased), and 124 differential metabolites between Middle and Mature (102 increased, 22 decreased). The most pronounced changes occurred in lipid metabolites, particularly triglycerides and phosphatidylcholines. KEGG enrichment analysis revealed that these differential metabolites were mainly involved in glycerolipid metabolism, glycerophospholipid metabolism, and insulin resistance pathways. Correlation network analysis further identified key genes associated with these metabolites, highlighting the interplay between lipid and amino acid metabolism during adipogenesis.

**Conclusion:**

This study provides a comprehensive metabolomic profile of adipocyte differentiation in 3T3-L1 cells, revealing significant alterations in lipid metabolism and key metabolic pathways. These findings enhance our understanding of the molecular mechanisms underlying adipogenesis and may contribute to the development of novel diagnostic tools and therapeutic strategies for obesity-related metabolic diseases.

## 1 Introduction

Obesity is widely recognized as a major risk factor for various metabolic diseases. Obesity can lead to increased cardiac workload and blood pressure, impairment of lung function, greater mechanical stress on weight-bearing joints, immune dysregulation with chronic low-grade inflammation, and alterations in endocrine signaling and metabolic homeostasis (Conway and Rene, 2004), all of which will impose a great burden on the body. More importantly, obese patients are also more troubled by hypertension, dyslipidemia, type 2 diabetes, coronary heart disease, stroke, gallbladder diseases, osteoarthritis, sleep apnea and respiratory problems, and even certain cancers, which increases the risk of cardiovascular diseases and all-cause mortality (Jensen et al., 2013). Obesity plays a central role in the development of these diseases (Pasquali et al., 2020; Abenavoli et al., 2019). Given its widespread health impacts, understanding the mechanisms behind obesity is essential for developing effective interventions. The global prevalence of obesity continues to rise sharply. As of 2022, more than one billion people worldwide were living with obesity (NCD Risk Factor Collaboration, 2024). This increase affects all age groups and socioeconomic levels, with the most rapid rises observed in high-income countries and in urban areas of low- and middle-income nations (Martinez and Grant, 2025). Consequently, there is an urgent need to formulate and implement effective strategies for the prevention and treatment of obesity. In this study, we employed the 3T3-L1 differentiation protocol, which induces a well-characterized sequence of cellular events: growth arrest and clonal expansion (approximately days 0–2), followed by growth arrest and the initiation of terminal differentiation marked by lipid accumulation and adipogenic gene expression (days 2–6), culminating in a mature adipocyte phenotype (day 8 onwards) (Sun et al., 2020; Sun et al., 2016). To capture the metabolomic shifts associated with these critical transitions, we sampled cells at three representative stages: pre-differentiation (Day 0, Pre), an intermediate point of differentiation (Day 4, Middle), and the fully mature state (Day 8, Mature).

Building on this epidemiological backdrop, it is important to define the condition in molecular and clinical terms. Obesity is a complex metabolic disease mainly characterized by abnormal or excessive accumulation of fat, posing a serious threat to human health (Schwartz et al., 2017). At the mechanistic level, the occurrence of obesity is related to multiple factors, including genetic factors and environmental factors and their interactions (van Baak and Mariman, 2019). Specifically, obesity is driven by the excessive proliferation of pre-adipocytes and the pronounced accumulation of lipids in mature adipocytes, both of which reflect an energy imbalance in which caloric intake exceeds expenditure. The surplus energy is stored as triglycerides, leading to an increase in both adipocyte size (hypertrophy) and number (hyperplasia).

Adipogenesis is the core process of adipose tissue development and energy metabolism regulation, and its abnormalities are closely related to metabolic diseases such as obesity and diabetes. Multiple studies have confirmed that obesity is closely related to the process of differentiation into adipocytes (Rosen and Spiegelman, 2006; Kim S. H. et al., 2008). Adipogenesis is the transformation of fibroblast into adipocytes from preadipocyte. A multi-phase process was followed in adipogenesis. Depending on the adipogenesis level, the expression pattern of transcripts and protein involved in adipogenesis was organized. A large body of research has demonstrated that a variety of regulatory factors—including circulating hormones, transcription factors, and growth factors—govern adipogenesis (Moseti et al., 2016). Key examples include peroxisome proliferator-activated receptor γ (Fève, 2005), CCAAT/enhancer-binding proteins (Mueller, 2014), fatty-acid-binding protein 4 (Cristancho and Lazar, 2011), fatty-acid synthase and glucose transporter type 4 (Amber-Vitos et al., 2016), as well as perilipin and other genes encoding lipid-droplet-associated proteins (Kim Y. J. et al., 2008). Therefore, a deeper understanding of adipocyte differentiation is not only scientifically important but also clinically relevant for combating obesity and its associated diseases.

Previous studies have shown that the molecular mechanisms of adipocyte differentiation and its regulation are very complex. Many transcription factors and metabolic enzymes play a role in the process of fibroblast differentiation into adipocytes. However, the different stages of adipocyte differentiation and their regulatory factors are not yet fully understood, and many links are still missing at present. Metabolomics provides a new perspective for revealing the mechanism of adipogenesis by systematically analyzing the dynamic changes of metabolites during cell differentiation. Notably, while individual metabolic changes during 3T3-L1 differentiation have been reported, a time-resolved metabolomic atlas integrated with transcriptomic data has not been systematically established. This study therefore aims to provide both a temporal metabolic landscape and a first integrated analysis of gene-metabolite networks across differentiation stages. This study will comprehensively reveal the metabolomic changes during the differentiation process of adipocytes.

## 2 Materials and methods

### 2.1 Sample information

To explore the differences of metabolites in 3T3-L1 cell, the sample groups and corresponding information are as follows.

**Table:** 

Tissue location	Treatment	Sample name	Group
3T3-L1 cell	Pre-adipocytes 1	Pre 1	Pre
3T3-L1 cell	Pre-adipocytes 2	Pre 2	Pre
3T3-L1 cell	Pre-adipocytes 3	Pre 3	Pre
3T3-L1 cell	Pre-adipocytes 4	Pre 4	Pre
3T3-L1 cell	4-day differentiated adipocytes 1	Middle 1	Middle
3T3-L1 cell	4-day differentiated adipocytes 2	Middle 2	Middle
3T3-L1 cell	4-day differentiated adipocytes 3	Middle 3	Middle
3T3-L1 cell	4-day differentiated adipocytes 4	Middle 4	Middle
3T3-L1 cell	8-day differentiated adipocytes 1	Mature 1	Mature
3T3-L1 cell	8-day differentiated adipocytes 2	Mature 2	Mature
3T3-L1 cell	8-day differentiated adipocytes 3	Mature 3	Mature
3T3-L1 cell	8-day differentiated adipocytes 4	Mature 4	Mature

### 2.2 Cell culture and differentiation

**FIGURE 1:**
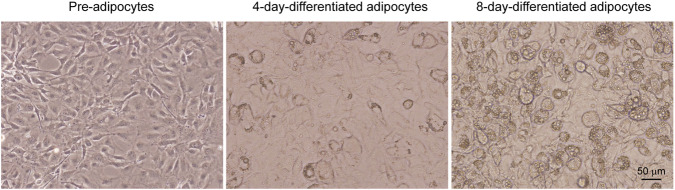
Representative morphological images of 3T3-L1 cells Representative morphological images of 3T3-L1 cells captured at three distinct stages: undifferentiated preadipocytes, cells at 4 days post-differentiation induction, and fully differentiated mature adipocytes at 8 days. Scale bar = 50 μm.

Murine 3T3-L1 preadipocytes were cultured and differentiated as previously described (Sun et al., 2020). Cells were seeded in 6-well plates and maintained in complete high-glucose Dulbecco’s Modified Eagle medium (DMEM; Gibco) supplemented with 10% fetal bovine serum (Gibco), 2 mM glutamine, 100 U/mL penicillin, and 100 μg/mL streptomycin. Differentiation was induced at 95% confluence (designated Day 0) by incubating cells for 48 h in induction medium consisting of complete medium containing 2 μg/mL dexamethasone, 0.5 mM 3-isobutyl-1-methylxanthine, and 10 μg/mL insulin. The medium was then changed to maintenance medium (complete medium with 10 μg/mL insulin) on Day 2, and fresh maintenance medium was refreshed on Day 4. Cells were cultured for a total of 8 days post-induction, achieving full differentiation into mature adipocytes (Figure 1). Cells were harvested at three time points: pre-differentiation, 4 days post-induction, and 8 days post-induction. Immediately after collection, cell pellets were snap-frozen in liquid nitrogen and stored at −80 °C.

### 2.3 Sample preparation and LC-MS/MS analysis

#### 2.3.1 Metabolite extraction

##### 2.3.1.1 Hydrophilic metabolite extraction

Cell pellets were thawed on ice, and 1 mL of pre-cooled 70% aqueous methanol was added. Samples were vortexed for 1 min, followed by three cycles of freezing in liquid nitrogen (3 min) and thawing on ice (3 min) with vortexing (2 min) after each thaw. The mixture was centrifuged at 12,000 × g for 10 min at 4 °C. The supernatant was collected and subjected to liquid chromatography–tandem mass spectrometry (LC-MS/MS) analysis.

##### 2.3.1.2 Hydrophobic (lipid) metabolite extraction

Cell pellets were subjected to rapid freezing in liquid nitrogen for 2 min, thawed on ice for 5 min, and vortexed. Samples were then centrifuged at 12,000 × g for 10 min at 4 °C. A 300 µL aliquot of the supernatant was mixed with 1 mL of a methanol/methyl tert-butyl ether (MTBE) solution containing internal standards. The mixture was vortexed for 2 min, 500 µL of water was added, and vortexing was continued for 1 min. After centrifugation (12,000 × g, 10 min, 4 °C), 500 µL of the upper organic layer was collected, dried under vacuum, and reconstituted in 100 µL of mobile phase B. Reconstituted samples were stored at −80 °C until LC-MS/MS analysis. Extraction efficiency and reproducibility were assessed using pooled quality control samples. Recovery rates for representative metabolites ranged from 88% to 112%, with intra- and inter-day relative standard deviations (RSDs) below 15%.

#### 2.3.2 Ultra-performance liquid chromatography conditions

Metabolite separation was performed on an LC-ESI-MS/MS system consisting of a Shimadzu UPLC and a SCIEX QTRAP® mass spectrometer.

##### 2.3.2.1 Hydrophilic metabolite separation

A Waters ACQUITY UPLC HSS T3 C18 column (1.8 µm, 2.1 × 100 mm) was used at 40 °C. The mobile phase consisted of 0.1% (v/v) formic acid in water and 0.1% (v/v) formic acid in acetonitrile. The flow rate was 0.4 mL/min with an injection volume of 2 µL.

##### 2.3.2.2 Hydrophobic metabolite separation

A Thermo C30 column (2.6 µm, 2.1 × 100 mm) was used at 45 °C. The mobile phase consisted of acetonitrile/water (60:40, v/v) with 0.04% acetic acid and 5 mmol/L ammonium formate, and acetonitrile/isopropanol (10:90, v/v) with 0.04% acetic acid and 5 mmol/L ammonium formate. The flow rate was 0.35 mL/min with an injection volume of 2 µL.

#### 2.3.3 ESI-QTRAP-MS/MS analysis

Mass spectrometric detection was performed in both positive and negative ionization modes using a QTRAP® 6500+ system (SCIEX) equipped with an ESI Turbo Ion-Spray source and controlled by Analyst® 1.6.3 software.

##### 2.3.3.1 Hydrophilic metabolite detection

ESI source conditions were as follows: source temperature, 500 °C; ion spray voltage, +5500 V (positive) and −4500 V (negative); ion source gas 1 (GS1), 55 psi; ion source gas 2 (GS2), 60 psi; curtain gas (CUR), 25 psi; collision gas (CAD), high. The instrument was tuned and calibrated using 10 and 100 μmol/L polypropylene glycol solutions in QQQ and LIT modes, respectively. A scheduled multiple reaction monitoring (MRM) method was employed, with transitions monitored according to metabolite-specific retention times.

##### 2.3.3.2 Hydrophobic metabolite detection

ESI source conditions were as follows: ion source, Turbo Spray™; source temperature, 550 °C; ion spray voltage, +5500 V; GS1, 55 psi; GS2, 60 psi; CUR, 25 psi; CAD, medium. Tuning and calibration were performed as described above. MRM scans were acquired with collision gas (nitrogen) pressure set to 5 psi. Declustering potential (DP) and collision energy (CE) were optimized for each MRM transition. A scheduled MRM method was used, monitoring transitions within specific time windows based on metabolite elution profiles.

### 2.4 RNA sequence and analysis

Total RNA extracted from adipocytes at distinct differentiation stages was pooled and forwarded to BGI (Shenzhen, China) for cDNA library construction and Illumina HiSeq 3000 paired-end sequencing (≈6 Gb raw reads per sample). After quality filtering with Trimmomatic, clean reads were aligned to the mouse reference genome (GRCm38) with HISAT2. Transcript abundance was quantified by RSEM; differential expression was determined by NOISeq and PoissonDis algorithms. Functional enrichment (DAVID; KOBAS 3.0) employed hypergeometric tests with Benjamini–Hochberg FDR correction. Data are available at GEO under accession GSE129957, as reported in our previous work (Sun et al., 2020).

### 2.5 Statistical analysis

The data were analyzed for significance difference using SPSS 25 statistical software. The data between the two groups were compared. If they conformed to the normal distribution, the Least Significant Difference test was used; if they did not conform to the normal distribution, the non-parametric Mann-Whitney U test was used. When comparing data among multiple groups, one-way analysis of variance was used if the variances were homogeneous. If the variances were not homogeneous, Dunnett’s T3 method was used for the test.

The differential metabolites were further screened out by combining the P-threshold or folding change value of univariate analysis. Principal component analysis (PCA) and orthogonal partial least squares discriminant analysis (OPLS-DA) were performed on the identified metabolites using R software. Significant differential metabolites were screened based on the variable importance projection (VIP) score obtained by the OPLS-DA model. Metabolites with VIP value >1 and P value <0.05 were defined as differential metabolites. The metabolic pathways of differential metabolites were annotated through the Kyoto Encyclopedia of Genes and Genomes (KEGG) database to obtain the metabolic pathways of differential metabolites.

## 3 Results

### 3.1 Sample quality control analysis

**FIGURE 2:**
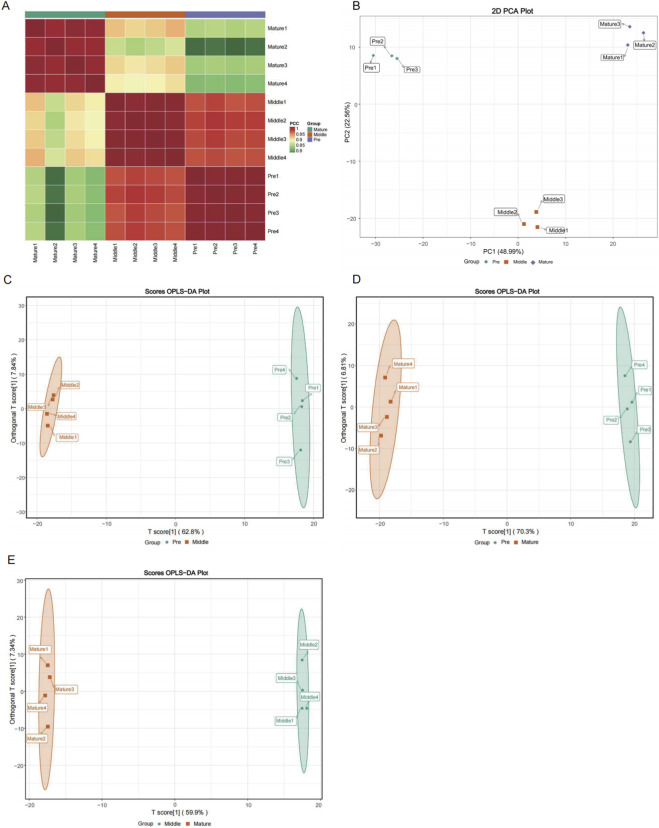
Sample quality control analysis **(A)**. Inter-sample correlation heatmap. **(B)** PCA score plot. Each point corresponds to a sample, and the distance between two points is related to the difference in the composition structure of metabolites in the sample. Different groups are marked with different colors. **(C-E)** OPLS-DA score plots (Pre vs. Middle, Pre vs. Mature, Middle vs. Mature).

To assess the reproducibility of biological replicates, Pearson correlation coefficient (PCC) was employed to evaluate the correlation among samples within each group. A PCC value closer to 1 indicates a stronger correlation between replicate samples. Higher intra-group correlation compared to inter-group correlation suggests reliable identification of differential metabolites (Figure 2A). PCA revealed clear separation between groups and high consistency within groups, indicating distinguishable metabolic profiles among the three differentiation stages (Figure 2B). OPLS-DA further demonstrated distinct clustering of samples from different groups, supporting significant metabolic differences between groups (Figures 2C–E).

### 3.2 Analysis of differential metabolites

**FIGURE 3:**
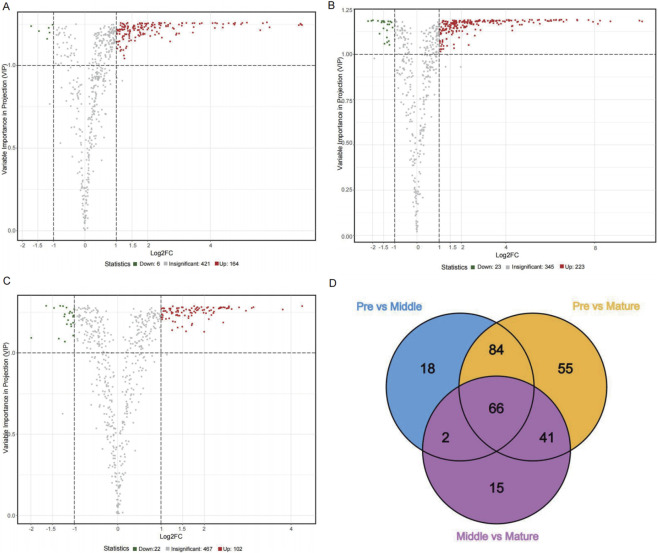
Analysis of differential metabolites **(A-C)** Volcano plots of differential metabolites. The green dots represent significantly decreased differential metabolites, the red dots represent significantly increased differential metabolites, and the gray dots represent detected metabolites with no significant differences. AuthorAnonymous, **(D)** Venn diagram of shared differential metabolites.

As shown in Figures 3A–C, the analysis of differential metabolites between pairs of the three groups of cells indicated that there was a total of 170 differential metabolites (164 increased and 6 decreased) in Pre and Middle. There was a total of 246 differential metabolites (223 increased and 23 decreased) in Pre and Mature. There was a total of 124 differential metabolites (102 increased and 22 decreased) in Middle and Mature. Venn diagram analysis identified 66 metabolites common to all three groups, predominantly lipid species including free fatty acids (FFA), phosphatidylethanolamines (PE), diacylglycerols (DG), phosphatidylcholines (PC), and triglycerides (TG) (Figure 3D). These results indicate extensive metabolic reprogramming occurs throughout adipocyte differentiation.

### 3.3 Fold change analysis of differential metabolites

**FIGURE 4:**
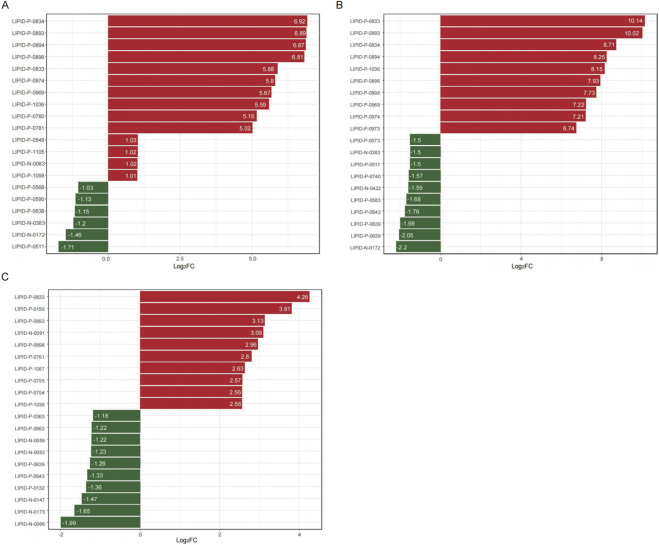
Fold change analysis of differential metabolites. **(A-C)** Bar charts showing fold changes of the top significantly altered metabolites (fold change >2, VIP >1, *p* < 0.05) for comparisons **(A)** Pre vs. Middle, **(B)** Pre vs. Mature, and **(C)** Middle vs. Mature. Specific metabolite names are provided in Supplementary Table S1.

Further difference multiple analysis was conducted on the differential metabolites. Significant fold changes (>2-fold) were observed for specific metabolites (Figure 4; Supplementary Table S1):

Pre vs. Middle: 10 differential metabolites exceeding 2-fold change. TG showed the most significant increase, while PC showed the most significant decrease (Figure 4A; Supplementary Table S1).

Pre vs. Mature: 12 differential metabolites exceeding 2-fold change. TG showed the most significant increase, while phosphatidic acid (PA) showed the most significant decrease (Figure 4B; Supplementary Table S1).

Middle vs. Mature: 10 differential metabolites exceeding 2-fold change. TG showed the most significant increase, while 6-keto-prostaglandin F1α showed the most significant decrease (Figure 4C; Supplementary Table S1).

### 3.4 KEGG pathway enrichment analysis of differential metabolites

**FIGURE 5:**
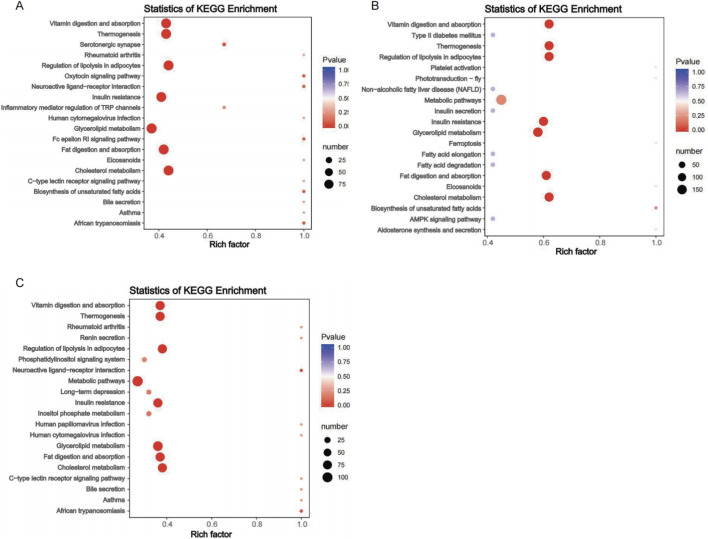
KEGG pathway enrichment analysis of differential metabolites. **(A-C)** KEGG enrichment bubble plots for each comparison group. The vertical axis represents the name of the KEGG metabolic pathway, and the horizontal axis represents the rich factor corresponding to each pathway. The color of the point is the pvalue, and the redder the point is, the more significant the enrichment is. The size of the point represents the number of differential metabolites enriched.

To explore the functions of differential metabolites among the three groups of cells, KEGG pathway enrichment analysis was conducted. The results showed that these metabolites were significantly enriched in pathways related to lipid metabolism, including vitamin digestion and absorption, thermogenesis, regulation of lipolysis in adipocytes, cholesterol metabolism, fat digestion and absorption, glycerolipid metabolism, glycerophospholipid metabolism, and insulin resistance (Figure 5). These pathways are closely associated with adipocyte differentiation and lipid homeostasis.

### 3.5 Trend Analysis of Metabolites During Differentiation

**FIGURE 6:**
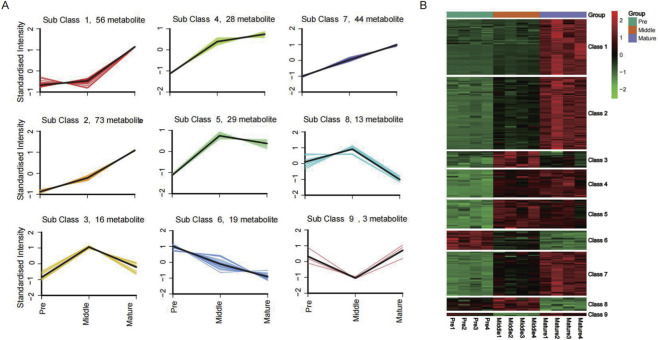
Trend Analysis of Metabolites During Differentiation **(A)**. Schematic diagram of cluster analysis of differential metabolites. The number at the bottom of each cluster indicates the quantity of differential metabolites within that cluster; **(B)** K-means metabolic heat maps of metabolite expression in cells at different stages. The color annotations of the heat map are displayed in the bottom color scale. Increased or decreased metabolites are indicated by red or green bars.

To study the changing trends of the relative contents of metabolites in cells at different stages, K-Means analysis was used to cluster the relative contents of all the screened differential metabolites. These screened differential metabolites had nine different changing trends during the differentiation process (Figure 6; Supplementary Table S2). Clusters 1, 2, 4, and 7 showed a continuous increase trend, including 201 metabolites such as FFA, PE, phosphatidylinositols (PI), DG, phosphatidylserines (PS), TG, ceramides (Cer), lysophosphatidylethanolamines (LPE), PC, phosphatidylglycerols (PG), TG esterified with FFA (TGFFA), lysophosphatidylcholines (LPC), and 3(S),6(R)-DiHETE. Cluster 6 exhibited a continuous decrease trend, including 19 metabolites such as PA, PE, cholesteryl esters (CE), Cer, DG, LPE, PC, and sphingomyelins (SM). Clusters 3, 5, and 8 displayed a pattern of initial increase followed by decrease, including 58 metabolites such as prostaglandins (PGJ2, PGD2, PGE2, PGD1), Coenzyme Q10, LPC, LPE, PC, TG esterified with PC (TGPC), PG, PE, TG esterified with prostaglandin E1 (TGPGE1), 6-keto-PGF1α, FFA, PA, Cer and DG. Cluster 9 showed a pattern of initial decrease followed by increase, including 3 PE-related metabolites. In conclusion, the changes of the nine types of differential metabolites in the three groups were all different during the differentiation process.

### 3.6 Integration of transcriptomic and metabolomic data

**FIGURE 7:**
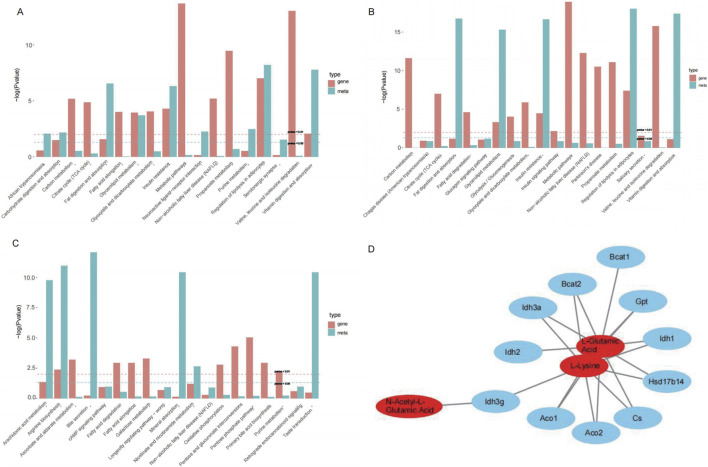
Integration of transcriptomic and metabolomic data. **(A-C)** KEGG enrichment bar charts. The horizontal axis of the bar chart represents the metabolic pathway. In the vertical axis, red represents the enriched p-value of the differentially expressed genes, and green represents the enriched P-value of the differentially expressed metabolites, denoted as -log (p-value). The higher the vertical axis, the stronger the degree of enrichment. **(D)** Correlation network of metabolites and genes in ko01210 pathway. Metabolites are marked in red and genes in blue, and the correlations between them are represented by straight lines.

Transcriptome sequencing reveals the differences in gene expression in specific tissues, while metabolomics clarifies the metabolic processes and change mechanisms of biological samples. The combined analysis of the two can more reasonably and accurately explain the potential regulatory mechanisms of phenotypes. To explore the regulatory mechanisms underlying adipocyte differentiation, differentially expressed genes and metabolites were co-analyzed using KEGG pathway enrichment. Notably, both gene and metabolite datasets were enriched in insulin resistance, glycerolipid metabolism, and regulation of lipolysis in adipocytes (Figures 7A–C).

Pathway ko01210 (insulin resistance) was selected as it showed the highest enrichment score in both transcriptomic and metabolomic datasets to analyze the correlation between metabolites and genes (Figure 7D). Additional pathways, including fatty acid biosynthesis (ko00061) and glycerolipid metabolism (ko00561), were also enriched. The results showed that L-glutamic acid is an important amino acid that participates in various metabolic processes in the body, such as nitrogen metabolism and energy metabolism. L-lysine plays an important role in protein synthesis, collagen formation, and the production of hormones and enzymes. N-acetyl-L-glutamic acid acts as an allosteric activator of carbamoyl phosphate synthase I in the urea cycle and participates in the detoxification of ammonia and the production of urea in the body. The isocitrate dehydrogenase (Idh) series (Idh1, Idh2, Idh3a, Idh3g) are isocitrate dehydrogenase genes and are involved in metabolic pathways such as the tricarboxylic acid cycle; aconitase (Aco1) and Aco2 encode acetyl-CoA oxidase, which is involved in the β-oxidation process of fatty acids and is a key enzyme in fatty acid metabolism.Branched-chain aminotransferase (Bcat1) and Bcat2 are branched-chain amino acid transaminase genes and are involved in branched-chain amino acid metabolism. Glutamate–pyruvate transaminase (Gpt) is the alanine aminotransferase gene and is involved in amino acid metabolism. 17β-hydroxysteroid dehydrogenase type 14 (Hsd17b14) is the 17β -hydroxysteroid dehydrogenase type 14 gene and is involved in steroid hormone metabolism. Citrate synthase (Cs) encodes citrate synthase, which participates in the tricarboxylic acid cycle and is an intermediate product of fatty acid metabolism. These genes may affect energy metabolism, fatty acid synthesis and metabolic regulation through amino acid metabolism.

## 4 Discussion

Obesity is characterized by abnormal expansion of white adipose tissue, mainly through hypertrophy (enlargement of existing adipocytes) or hyperplasia (differentiation of preadipocytes into mature adipocytes) (Ghaben and Scherer, 2019). Thus, controlling preadipocyte proliferation is an effective strategy for obesity prevention or treatment (Choi et al., 2023). The 3T3-L1 mouse preadipocyte line is widely used to study adipogenesis (Cao et al., 2023). This study investigated metabolomic changes during 3T3-L1 differentiation to uncover molecular mechanisms underlying adipogenesis and obesity-related metabolic diseases. Our findings reveal dynamic metabolic shifts from preadipocytes to mature adipocytes, offering insights into potential therapeutic targets. Compared to previous metabolomic studies, this work provides time-resolved multi-omics integration, highlighting stage-specific metabolic changes and identifying novel gene–metabolite networks, especially in amino acid metabolism, an area understudied in this context.

Preadipocyte differentiation is a complex, orderly process divided into prophase, middle phase, and late phase (Koutnikova and Auwerx, 2001). We obtained transcriptomic and metabolomic data from 3T3-L1 cells at distinct stages using RNA-seq and LC-MS/MS. Sample correlation confirmed experimental reliability. PCA showed clear separation among the three stages (Figure 2B), and OPLS-DA further demonstrated robust metabolic differences (Figures 2C–E). Our metabolomic analysis revealed significant differences in metabolite profiles among Pre, 4-day differentiated (Middle), and 8-day mature (Mature) adipocytes. Volcano plots and Venn diagrams (Figures 3, 4) showed extensive alterations, particularly in lipid classes such as FFA, PE, DG, PC, and TG. This highlights profound lipid metabolic reprogramming during differentiation, consistent with lipid accumulation as a hallmark of adipocyte maturation (Zhang et al., 2016). The marked increase in TG and decrease in PC from Pre to Middle and Pre to Mature (Figure 6) reflect a shift toward lipid storage and membrane remodeling (Zadoorian et al., 2023). The progressive nature of these changes underscores the complexity and coordinated regulation of adipogenesis. KEGG enrichment analysis pinpointed key altered pathways, including glycerolipid metabolism, glycerophospholipid metabolism, and insulin resistance (Figure 5). These pathways are crucial for energy homeostasis and adipocyte function. Glycerolipid enrichment indicates active synthesis (for storage) and hydrolysis (for signaling) of TG and DG as adipocytes mature. Glycerophospholipid enrichment relates to biosynthesis and turnover of membrane phospholipids (PC, PE, PS), required for structural remodeling during cell enlargement and lipid droplet formation (Zadoorian et al., 2023). The insulin resistance pathway association is significant: intrinsic metabolic changes during adipogenesis may contribute to systemic insulin resistance in obesity, increasing susceptibility to type 2 diabetes (Ruze et al., 2023).

Beyond known pathways, our analysis reveals crosstalk between amino acid and lipid metabolism. The correlation of L-glutamic acid with BCAT1/2 and IDH genes suggests that branched-chain amino acid (BCAA) catabolism fuels lipid synthesis, a mechanism not systematically mapped in time-resolved 3T3-L1 differentiation. Additionally, the biphasic pattern of prostaglandins (PGJ2, PGD2, PGE2) in Cluster 3 (Figure 6) suggests transient regulatory roles in mid-differentiation, unreported previously. Moreover, the specific decrease in PC species coupled with increases in PE, PS, and PI indicates coordinated phospholipid remodeling required for lipid droplet biogenesis, beyond simple triglyceride accumulation. The correlation network linked differentially expressed genes and metabolites, providing a holistic view of regulatory mechanisms. Genes such as *Idh* series, *Aco1*, *Aco2*, *Bcat1*, *Bcat2*, *Gpt*, and *Hsd17b14* were closely associated with metabolites like L-glutamic acid, L-lysine, and N-acetyl-L-glutamic acid (Figure 7). These genes are involved in the TCA cycle, BCAA metabolism, and steroid hormone metabolism. Their interplay suggests that amino acid metabolism and energy production support adipocyte differentiation. *Idh* genes encode isocitrate dehydrogenases, critical for generating NADPH and ATP required for lipid synthesis and cell proliferation (Parsons et al., 2008). *Bcat* genes are involved in BCAA catabolism, providing precursors for gluconeogenesis and energy production (Green et al., 2016; Tönjes et al., 2013). The correlation between L-glutamic acid and related genes indicates tight interplay between amino acid and lipid metabolism (Masoodi et al., 2021). L-glutamic acid is a key nitrogen metabolite that converts to α-ketoglutarate, a central TCA cycle intermediate. This connection suggests that during differentiation, cellular metabolism reorients to prioritize lipid synthesis and storage, with amino acid metabolism providing precursors and energy. N-acetyl-L-glutamic acid, an activator of carbamoyl phosphate synthase I in the urea cycle, may reflect efforts to detoxify ammonia and maintain nitrogen balance as metabolic activity intensifies (Zhao et al., 2022). The metabolomic changes identified may have significant implications for managing obesity-related diseases. Differential metabolites and enriched pathways could serve as biomarkers for adipogenesis and obesity progression. For example, increased TG and decreased PC may indicate increased lipid storage and altered membrane composition, potentially preceding insulin resistance (Niu et al., 2023). Tracking these metabolites could help assess obesity-related disease risk and enable early intervention.

Moreover, these findings may guide novel therapeutic strategies targeting specific metabolic pathways in adipogenesis. Modulating enzymes or transporters in glycerolipid or glycerophospholipid metabolism could inhibit excessive lipid accumulation (Wang et al., 2022). Targeting key genes and metabolites in insulin resistance pathways might improve insulin sensitivity and reduce the metabolic burden of obesity (Wang et al., 2023). However, further research is needed to validate these potential targets and explore their safety and efficacy clinically. Our findings in differentiating 3T3-L1 cells show striking parallels with clinical lipidomic profiles from obese individuals. Elevated serum triglycerides and altered PC species (decreased PC (34:1) and PC (36:1)) have been reported in obese patients *versus* lean controls (Gohda et al., 2023). Similarly, our data show increased TG and decreased PC during differentiation (Figures 4A,B), suggesting the PC/TG ratio may serve as a cellular correlate of adipose tissue dysfunction. Furthermore, BCAA levels are elevated in obesity and insulin resistance, and our integrated analysis links BCAA catabolism (BCAT1/2) directly to lipid synthesis. This convergence supports the translational relevance of our findings and suggests that adipose tissue BCAT activity might contribute to circulating BCAA levels in obesity. Future clinical studies should explore whether plasma PC/TG ratios or BCAA levels correlate with adipose tissue differentiation status in human obesity.

Abnormal lipid metabolism is not merely a storage phenomenon—excessive or dysregulated lipid accumulation can trigger lipotoxicity and cell death. Zadoorian et al. (Wu et al., 2023) showed that impaired lipid droplet biogenesis leads to accumulation of toxic lipid intermediates (e.g., ceramides, DAG), inducing ER stress and apoptosis. Similarly, metabolic inflexibility in adipocytes has been linked to ferroptosis, an iron-dependent cell death pathway triggered by lipid peroxidation (Wu et al., 2022). Although our study did not directly assess cell death, the dynamic changes in phospholipids (PC, PE, PS) and the transient increase in prostaglandins (which modulate oxidative stress) may reflect adaptive mechanisms to prevent lipotoxicity. Future studies should examine whether the metabolic shifts we identified, particularly the PC-to-TG transition, represent a protective mechanism against lipid-induced cell death or, when dysregulated, a predisposition to adipocyte dysfunction and death.

Glycerolipid and glycerophospholipid metabolism reflect two intertwined demands during adipogenesis: energy storage and membrane remodeling. The marked increase in triglycerides (TG, storage lipids) and decrease in phosphatidylcholine (PC, a membrane phospholipid) from preadipocytes (Pre) to mature (Mature) stages (Figure 4; Supplementary Table S1) indicate a functional shift from maintaining plasma membrane integrity (high PC) to expanding lipid droplet surface area (requiring phospholipids like PE and PS). This aligns with Zadoorian et al., who showed that lipid droplet biogenesis requires coordinated phospholipid remodeling (Wu et al., 2023). Importantly, these changes are progressive, suggesting that membrane composition dynamically adapts to expanding lipid storage capacity. Dysregulation of this PC-to-TG transition may lead to lipotoxicity, as accumulation of membrane phospholipid intermediates is linked to ER stress and adipocyte dysfunction.

L-glutamic acid is a key intermediate in nitrogen metabolism and converts to α-ketoglutarate, a central TCA cycle metabolite. This connection supports cellular energy demands and anabolic processes during differentiation. As shown in our correlation network (Figure 7D), L-glutamic acid is linked to Idh1/2/3 and Bcat1/2, indicating that glutamic acid metabolism is tightly coordinated with BCAA catabolism and TCA cycle flux. L-glutamic acid can be converted to α-ketoglutarate, a TCA cycle intermediate that fuels ATP production and provides carbon skeletons for fatty acid synthesis. The concurrent upregulation of Idh genes (converting isocitrate to α-ketoglutarate) suggests a coordinated loop: glutamine/glutamate metabolism feeds into the TCA cycle, generating citrate for lipid synthesis. This mechanism operates in cancer cell proliferation and also in adipocyte differentiation (Kruk et al., 2015). Our data extend these findings by providing a temporal map of this crosstalk across differentiation stages. Interventions targeting BCAA catabolism (e.g., BCAT inhibitors) might simultaneously reduce lipid accumulation and improve insulin sensitivity, a dual therapeutic opportunity.

Several limitations should be acknowledged. First, the 3T3-L1 cell line may not fully recapitulate human adipose tissue. Future studies should validate key findings in human primary adipocytes or adipose tissue samples. *In vivo* studies in animal models and humans are necessary for clinical translation. Second, metabolomic analysis was performed at three time points; dynamic changes between these points may not have been fully captured. More frequent sampling could provide a finer temporal profile. Third, the correlation analysis between genes and metabolites was based on existing databases and bioinformatics tools; direct experimental validation is required. While our transcriptomic data were previously validated by qPCR for key adipogenic markers in our earlier work (Sun et al., 2020), the novel gene–metabolite correlations identified here (e.g., BCAT1/2 with L-glutamic acid; *IDH2* with N-acetyl-L-glutamic acid) need direct validation. Future studies involving genetic manipulation (e.g., siRNA knockdown or overexpression of *Bcat1*, *Bcat2*, or *Idh2*) coupled with targeted metabolomics are needed to confirm their regulatory roles in lipid–amino acid crosstalk. Additionally, pharmacological inhibition of BCAT activity (e.g., using gabapentin) could functionally test whether BCAA catabolism is required for lipid accumulation during adipogenesis.

## 5 Conclusion

This study provides a comprehensive time-resolved metabolomic analysis of adipocyte differentiation in 3T3-L1 cells, integrated with transcriptomic data to construct gene–metabolite networks. Two central pathway networks emerge as core mechanisms driving adipogenesis: (1) the glycerolipid and glycerophospholipid metabolism network, which orchestrates the dynamic shift from membrane phospholipids to storage triglycerides, involving specific phospholipid species (PE, PS, PI) required for lipid droplet expansion; and (2) the insulin resistance pathway integrated with amino acid metabolism, where BCAA catabolism (via BCAT1/2) and TCA cycle intermediates (via IDH genes) interface with lipid synthesis through L-glutamic acid and α-ketoglutarate. These interconnected networks highlight that adipogenesis is not merely lipid accumulation but a coordinated reprogramming of membrane architecture, energy metabolism, and amino acid catabolism. Our findings enhance the molecular understanding of adipogenesis and suggest potential biomarker candidates (e.g., PC/TG ratio, L-glutamic acid levels) and therapeutic targets (e.g., BCAT1/2, IDH2) for obesity-related metabolic diseases. Future studies should validate these targets in human primary adipocytes and animal models.

## Data Availability

The data used to support the findings of this study are available from the corresponding authors upon request.
