# Supplementary material for: Metabolomic profiling reveals dynamic lipid reprogramming during adipogenesis in 3T3-L1 cells
Source: Front Mol Biosci. 2026 May 26;13:1821798. doi: 10.3389/fmolb.2026.1821798 (PMC13246343; doi:10.3389/fmolb.2026.1821798)
Supplement: Supplementary file 1 [file DataSheet2.pdf]

Table 2. Different class of metabolites

Class	metabolites
Class 1	FFA, PE,PI,DG,PS,TG
Class 2	FFA,PE,Cer,DG,LPE,PC,PG,TG
Class 3	PGJ2,PGD2,PGE2,PGD1,Coenzyme Q10,LPC,LPE,PC,TG
Class 4	FFA,PC,PG,PS,Cer,LPC,TG
Class 5	PC,PG,LPC,LPE,PE,TG
Class 6	PA,PE,CE,Cer,DG,LPE,PC,SM
Class 7	3(S),6(R)-DiHETE,FFA,PC,PE,LPC,TG
Class 8	PGE1,6 keto-PGF1,FFA,LPE,PA,PC,Cer,DG,PE
Class 9	PE
