# Supplementary material for: Metabolomic profiling reveals dynamic lipid reprogramming during adipogenesis in 3T3-L1 cells
Source: Front Mol Biosci. 2026 May 26;13:1821798. doi: 10.3389/fmolb.2026.1821798 (PMC13246343; doi:10.3389/fmolb.2026.1821798)
Supplement: Supplementary file 2 [file DataSheet1.pdf]

Table 1: Details of differential metabolites

Index	Compounds	Class	Material category	Trend
Pre vs Middle				
LIPID-P-0833	TG(12:0/12:0/18:2)	TG	Triglyceride	↑
LIPID-P-0893	TG(12:0/14:1/18:2)	TG	Triglyceride	↑
LIPID-P-0834	TG(14:1/14:1/16:0)	TG	Triglyceride	↑
LIPID-P-0894	TG(14:1/14:1/18:1)	TG	Triglyceride	↑
LIPID-P-0896	TG(12:0/16:0/18:3)	TG	Triglyceride	↑
LIPID-P-0974	TG(14:1/14:1/20:2)	TG	Triglyceride	↑
LIPID-P-0969	TG(14:1/14:1/18:2)	TG	Triglyceride	↑
LIPID-P-0780	TG(12:0/16:0/16:1)	TG	Triglyceride	↑
LIPID-P-0548	PC(O-18:3/20:2)	PC-O	Phosphatidylcholine	↑
LIPID-P-1098	TG(16:0/16:1/22:5)	TG	Triglyceride	↑
LIPID-P-1105	TG(14:0/20:2/22:4)	TG	Triglyceride	↑
LIPID-N-0083	FFA(17:0)	FFA	Free fatty acids	↑
LIPID-P-0781	TG(12:0/14:0/18:1)	TG	Triglyceride	↑
LIPID-P-1036	TG(14:0/18:2/18:3)	TG	Triglyceride	↑
LIPID-P-0511	PC(O-12:0/14:0)	PC-O	Phosphatidylcholine	↓
LIPID-P-0638	PE(P-18:0/16:0)	PE-P	Phosphatidylethanolamine	↓
LIPID-N-0172	PA(16:0/20:1)	PA	Phosphatidic acid	↓
LIPID-N-0383	PE(20:1/16:0)	PE	Phosphatidylethanolamine	↓
LIPID-P-0590	PE(18:1/18:1)	PE	Phosphatidylethanolamine	↓
LIPID-P-0568	PE(16:0/16:0)	PE	Phosphatidylethanolamine	↓
Pre vs Mature				

LIPID-P-0833	TG(12:0/12:0/18:2)	TG	Triglyceride	↑
LIPID-P-0893	TG(12:0/14:1/18:2)	TG	Triglyceride	↑
LIPID-P-0898	TG(14:1/16:1/18:1)	TG	Triglyceride	↑
LIPID-P-1036	TG(14:0/18:2/18:3)	TG	Triglyceride	↑
LIPID-P-0834	TG(14:1/14:1/16:0)	TG	Triglyceride	↑
LIPID-P-0894	TG(14:1/14:1/18:1)	TG	Triglyceride	↑
LIPID-P-0896	TG(12:0/16:0/18:3)	TG	Triglyceride	↑
LIPID-P-0974	TG(14:1/14:1/20:2)	TG	Triglyceride	↑
LIPID-P-0973	TG(14:0/16:1/18:3)	TG	Triglyceride	↑
LIPID-P-0969	TG(14:1/14:1/18:2)	TG	Triglyceride	↑
LIPID-P-0639	PE(P-18:0/18:0)	PE-P	Phosphatidylethanolamine	↓
LIPID-P-0643	PE(P-18:1/18:0)	PE-P	Phosphatidylethanolamine	↓
LIPID-P-0073	Cer(d18:1/16:1)	Cer	Ceramide	↓
LIPID-P-0511	PC(O-12:0/14:0)	PC-O	Phosphatidylcholine	↓
LIPID-P-0583	PE(24:0/18:1)	PE	Phosphatidylethanolamine	↓
LIPID-P-0638	PE(P-18:0/16:0)	PE-P	Phosphatidylethanolamine	↓
LIPID-P-0740	SM(d18:1/26:1)	SM	Sphingomyelin	↓
LIPID-N-0172	PA(16:0/20:1)	PA	Phosphatidic acid	↓
LIPID-N-0383	PE(20:1/16:0)	PE	Phosphatidylethanolamine	↓
LIPID-N-0422	PE(20:1/18:1)	PE	Phosphatidylethanolamine	↓
Middle vs Mature				
LIPID-P-0833	TG(12:0/12:0/18:2)	TG	Triglyceride	↑

LIPID-P-0150	DG(16:1/16:1/0:0)	DG	Diglycerol ester	↑
LIPID-P-0893	TG(12:0/14:1/18:2)	TG	Triglyceride	↑
LIPID-P-0898	TG(14:1/16:1/18:1)	TG	Triglyceride	↑
LIPID-P-0761	TG(14:0/18:0/20:0)	TG	Triglyceride	↑
LIPID-P-1087	TG(14:0/18:2/20:4)	TG	Triglyceride	↑
LIPID-P-0755	TG(14:0/18:0/18:0)	TG	Triglyceride	↑
LIPID-P-0754	TG(16:0/16:0/18:0)	TG	Triglyceride	↑
LIPID-P-1036	TG(14:0/18:2/18:3)	TG	Triglyceride	↑
LIPID-N-0091	FFA(16:1)	FFA	Free fatty acids	↑
LIPID-P-0363	PC(20:1/22:1)	PC	Phosphatidylcholine	↓
LIPID-P-0663	PE(P-20:2/20:3)	PE-P	Phosphatidylethanolamine	↓
LIPID-P-0639	PE(P-18:0/18:0)	PE-P	Phosphatidylethanolamine	↓
LIPID-P-0643	PE(P-18:1/18:0)	PE-P	Phosphatidylethanolamine	↓
LIPID-P-0132	DG(14:1/16:0/0:0)	DG	Diglycerol ester	↓
LIPID-N-0059	PGE1	Eicosanoid	Arachidonic acid	↓
LIPID-N-0066	6 keto-PGF1α	Eicosanoid	Arachidonic acid	↓
LIPID-N-0053	PGE2	Eicosanoid	Arachidonic acid	↓
LIPID-N-0147	LPE(0:0/20:1)	LPE	Lysophosphatidylethanolamine	↓
LIPID-N-0173	PA(20:1/18:0)	PA	Phosphatidic acid	↓

Abbreviations: (↑), increase; (↓), decrease.
